# A simple method to rapidly assess the tibial tuberosity—trochlear groove distance using computed tomography

**DOI:** 10.1007/s11845-023-03302-z

**Published:** 2023-02-14

**Authors:** Simone L. Kneafsey, Shane P. Russell, Fiachra R. Power, Eric J. Heffernan, Conor Hurson

**Affiliations:** 1 Department of Trauma and Orthopaedics, St. Vincent’s University Hospital, Dublin, Ireland; 2 University College Dublin, Dublin, Ireland; 3 Department of Radiology, St. Vincent’s University Hospital, Dublin, Ireland

**Keywords:** Anatomical risk factors, Orthopaedics, Patellofemoral instability, Rapid assessment

## Abstract

**Background:**

The tibial tuberosity–trochlear groove (TTTG) distance is used to assess patellofemoral instability (PFI) and the likelihood of the development of patellofemoral disorders. The current gold standard in the assessment of the TTTG is computed tomography (CT) or magnetic resonance imaging (MRI). The current image software used for viewing these CT images does not allow for easy assessment of the TTTG.

**Aims:**

This study presents a simple method to measure the TTTG on any image software, utilizing easily available and affordable stationary.

**Methods:**

Four consecutive patients with no known knee pathologies were selected from recent studies at our institution. Their TTTGs were measured using this study’s method and validated using the standard, freely available image analysis software Fiji. Pre-defined anatomical landmarks were located and marked using adhesive pieces of paper. The TTTG was defined as the distance between parallel lines through the apex of the tibial tuberosity and trough of the trochlear groove, where each of these lines is perpendicular to the Dorsal Condylar Line.

**Results:**

The TTTG measured using this study’s method was found to be in agreement with the measurements made using Fiji software.

**Conclusions:**

This study demonstrates that the TTTG can be simply and quickly assessed using readily available and affordable stationery, without the need for expensive or complex secondary analysis software. This could allow for the assessment of PFI in the outpatient clinic whilst the patient is present, offering valuable assistance to the orthopaedic surgeon in clinical decision making.

## Introduction

The gold standard radiological assessment of the tibial tuberosity–trochlear groove (TTTG) distance, used in assessing lateral patellofemoral instability (PFI), is computed tomography (CT) or magnetic resonance imaging (MRI) measurement [1, 2]. The TTTG is the distance in the axial plane between the trough of the trochlear groove and the apex of the tibial tuberosity, with larger distances associated with increased PFI and resultant patellofemoral disorders. As per Schottle et al. patellofemoral disorders may be categorized as follows [3]:

- (i) Patellar instability with dislocation or subluxation associated with trochlear dysplasia.
- (ii) Patellar instability with dislocation or subluxation associated without trochlear dysplasia.
- (iii) Patellofemoral pain syndrome without dislocation, associated with trochlear dysplasia.
- (iv) Patellofemoral pain syndrome without anatomical abnormalities.
- (v) Isolated patellofemoral arthritis.

In 1994, Dejours et al. described four radiologically identified anatomical risk factors that were present in a higher frequency in patients with PFI in comparison to controls: trochlear dysplasia, patellar tilt with quadriceps dysplasia, patella alta and, most relevant to our study, an increased TTTG [4]. An increased TTTG contributes to the pathophysiology of PFI due to relative lateralization of the tibial tuberosity or medialisation of the trochlear groove [5, 6]. A pathologically increased TTTG may be an indication for a distal realignment procedure, such as medialisation of the tibial tubercle, to treat PFI [6]. As per Dejour et al. [4] a pathological TTTG is described as > 20 mm, with normal values being approximately 13 mm.

Many institutions utilise image-viewing software that does not have the capacity to easily measure the TTTG distance. As the tibial tubercle and trochlear groove lie in separate axial slices, image-viewing software is often incapable of measuring the coronal distance between these landmarks. Consequently, various techniques have been employed to obtain the TTTG, including the utilisation of expensive and/or complex secondary software. We propose a simplified method for TTTG measurement that (1) can be performed with standard viewing software, eliminating the need for secondary software analysis and (2) allows for the TTTG to be easily and rapidly assessed in any setting, including the busy out-patient clinic. We propose an accessible, uncomplicated measurement method that utilises standard, readily available office stationery, a concept previously demonstrated by Hughes et al. for measuring the rotational profile of total knee arthroplasty components [7].

## Materials and methods

### Patient details

CT scans were obtained of the right knee of four patients, two male and two female, ranging in ages from 32 to 81. These patients had presented to our institution following trauma between August 2021 and May 2022 where no history of knee pathologies was present, and no significant knee pathologies were subsequently identified.

### Imaging analysis

Knee CT imaging was reviewed by two authors (SK, SR). Using our novel method described below, the TTTG was first measured indirectly utilising three commonly used image viewing applications that do not have the capacity to measure TTTG directly: Syngo FastVIEW (Syngo, Siemens), Horizon Rad Station (McKesson Information Solutions) and RadAnt (RadAnt, Medixant).

### Validation of methodology

**Fig. 1:**
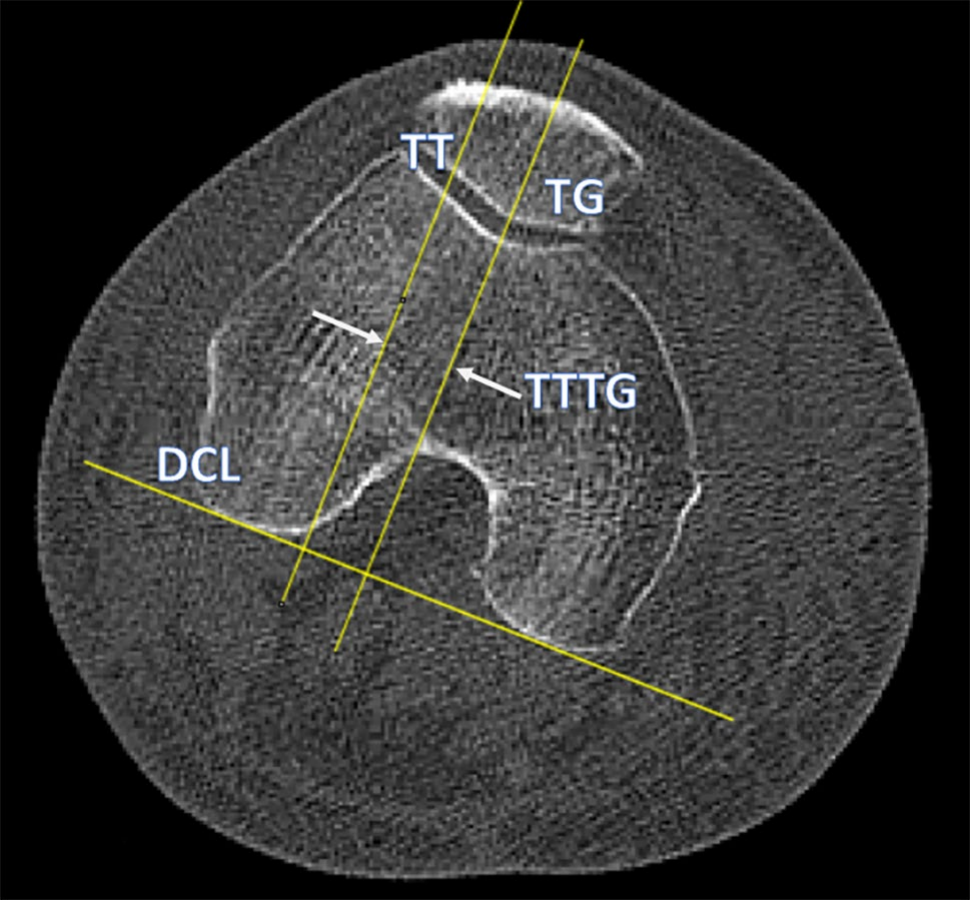
DCL, the most dorsal aspects of the medial and lateral femoral condyles; TG, the deepest part of the trochlear groove, the line drawn perpendicular to the DCL; TT, the most anterior part of the tibial tuberosity, perpendicular to the DCL and parallel with the TG; TTTG, the distance between TT and TG

Secondly, to validate the measurements obtained using our novel method, the TTTG was re-measured using Fiji software (open source) which has the capacity to measure TTTG directly [8] (Fig. 1).

### Measurement of the TTTG

**Fig. 2:**
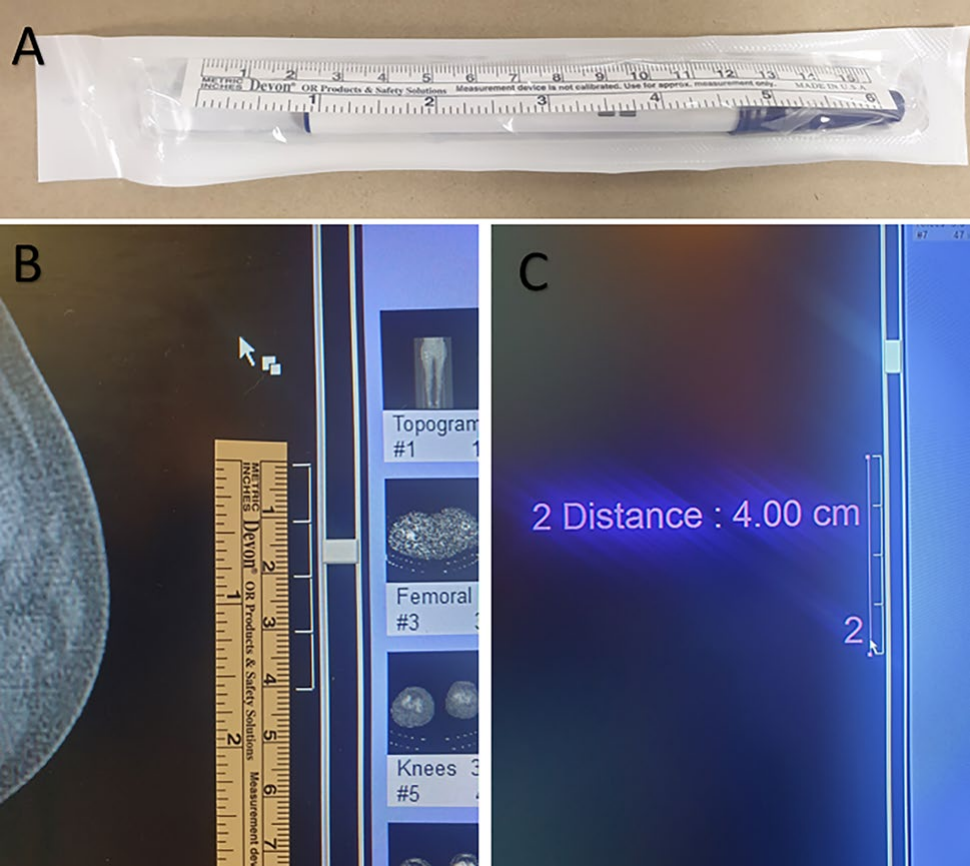
**A** A standard, easily available ruler, **B** calibration of imaging, **C** confirmation of correct calibration using Syngo FastVIEW

For the following described method, the TTTG is assessed using the technique described by Schoettle et al. [3]. Firstly, the scale bar of the axial CT image is calibrated to a standard ruler by adjusting the image zoom until the graduation marks of the scale bar correspond to the centimetre marks on the ruler (Fig. 2).

**Fig. 3:**
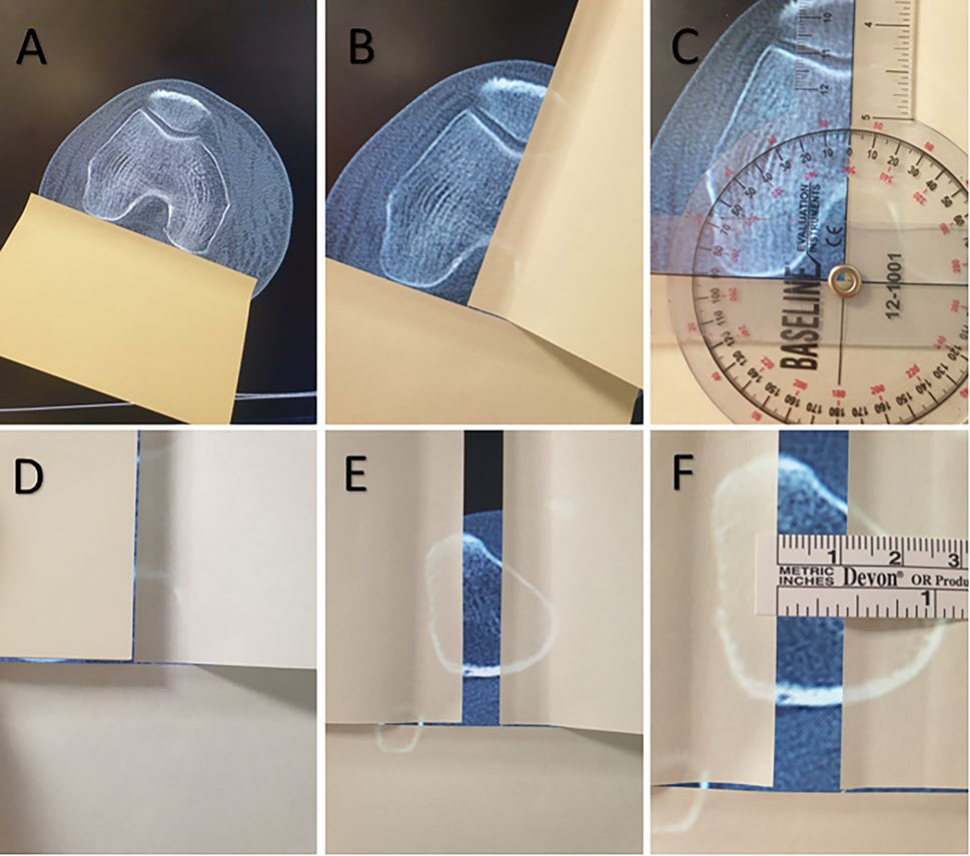
**A** The most dorsal aspects of the medial and lateral femoral condyles (DCL) located and marked with a sticky note, **B** the deepest part of the trochlear groove (TG) marked with a piece of adhesive paper, so as to be perpendicular to the DCL, **C** the DCL and TG lines are perpendicular as per a standard protractor measuring 90 degrees, **D** the TT line piece of adhesive paper positioned so as to be parallel with the TG line and perpendicular to the DCL line, **E** the most anterior part of the tibial tuberosity (TT) marked by moving the pre-positioned piece of adhesive paper out so as to remain perpendicular to the DCL and parallel with the TG, **F** the measurement of the TTTG (11 mm)

The dorsal condylar line (DCL) is then defined as the line between the most dorsal aspects of the medial and lateral condyles of the femur. This is marked by attaching an adhesive piece of paper (Post-It (3 M United Kingdom PLC, Berkshire, UK)) that is commonly found in outpatient clinics, to the monitor along the DCL (Fig. 3).

The axial CT image is then scrolled craniocaudally, leaving the piece of adhesive paper attached to the screen, until the deepest point of the trochlear groove (TG) is located. A second sticky note is placed on the monitor at this point so that it is perpendicular to the DCL, measured using a standard protractor (Fig. 3). Alternatively, any known right angle such as the corner of another note may be used.

Leaving both pieces of paper in situ, the CT scan is scrolled caudally until the most anterior point of the tibial tuberosity (TT) is reached. A third piece of adhesive paper is placed here so that it is perpendicular to the DCL sticky note and parallel to the TG sticky note (Fig. 3). The TTTG is measured as the distance between the two parallel pieces of adhesive paper, TT and TG (Fig. 3).

### Statistical analysis

The results were plotted on a Bland–Altman plot and assessed for the degree of bias using GraphPad Prism Software (version 9.0.0 for Windows, GraphPad Software, San Diego, CA, USA, www.graphpad.com).

## Results

The TTTG of four patients, without any relevant knee pathologies, were quickly and simply identified and measured using a standard ruler, stationery and image viewing software commonly found in an outpatient setting (see Table 1). This was repeated using three different viewing applications that do not allow for direct measurement.

**Table 1:** TTTG measurements of four patients without any known knee pathologies using a new novel method and validation with secondary analysis using Fiji software

Novel TTTG measurement method	Secondary analysis using Fiji	Difference
11 cm	10.97 cm	0.27%
11 cm	10.64 cm	3.38%
9 cm	9.44 cm	4.66%
17 cm	17.44 cm	2.52%

**Fig. 4:**
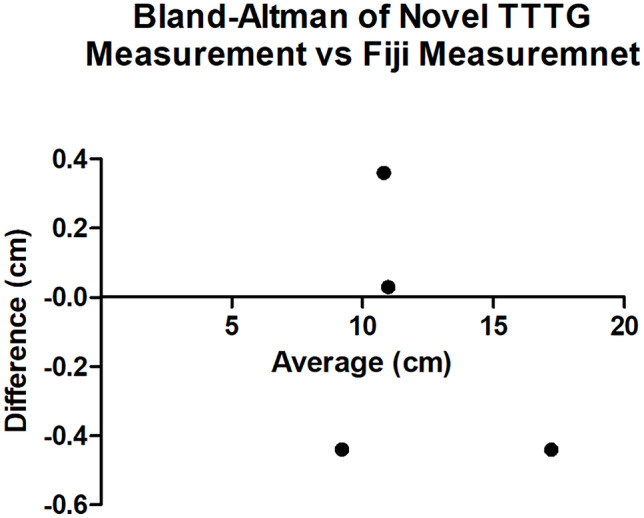
Bland–Altman Plot of differences between measurements using the novel TTTG method and Fiji method versus the average of the two measurements

Secondly, the TTTG was directly measured using Fiji software (see Table 1). These results were plotted on a Bland–Altman Plot (Fig. 4).

The bias between the two measurement methods was found to be − 0.12 cm (SD ± 0.39 cm) with a 95% limit of agreement of − 0.89 cm to 0.64 cm. The average percentage difference between measurement results was 0.88% (SD ± 3.02%).

## Discussion

CT images are easily accessed in the outpatient’s clinic and are a popular and widely available clinical investigation tool. The described technique allows for rapid assessment of the TTTG in the clinical setting, without the need to export images to secondary analysis software and enables risk factors for patellofemoral instability to be assessed using accessible, affordable everyday stationery equipment.

The TTTG is an accurate and reliable tool in the assessment of patellofemoral stability, with a TTTG of greater than 20 mm being considered a contributory cause of PFI. The ability to perform a rapid and simple assessment of the TTTG as a clinical decision-making tool would be of great benefit to orthopaedic surgeons worldwide. In addition, the ability to visually demonstrate a pathological TTTG, or lack thereof, to the patient at the point of consultation has the benefit of involving the patient in the decision-making process, allowing for the rationale of the surgeon to be understood.

The method was performed on three separate viewing software systems that are widely available in the outpatient clinic, each measuring the same value for the TTTG. It was validated by measuring the TTTG using Fiji software, with a bias between measurements of −0.12 cm (SD ± 0.39 cm) and an average difference of 0.88% (SD ± 3.02%) between results, which the authors find clinically insignificant.

This method has several limitations. As the dorsal condylar line, the most anterior point of the tibial tuberosity and the deepest point of the trochlear groove must be identified and marked using adhesive notes that are precisely perpendicular and parallel, this method is vulnerable to human error and interobserver variability. The use of standardised anatomical landmarks and methods for identifying these landmarks serves to mitigate and reduce such differences.

## Conclusion

The TTTG can be simply and quickly assessed in the outpatient clinic using affordable and readily available stationery equipment, without the need for secondary analysis software. The described method of TTTG measurement may offer valuable assistance to surgeons during the assessment of PFI.

## Data Availability

All data supporting the findings of this study are available within the article and raw data is available from the corresponding author, upon reasonable request.

## References

[1] Greiwe RM, Saifi C, Ahmad CS, Gardner TR (2010) Anatomy and biomechanics of patellar instability. Oper Tech Sports Med 18:62–67. 10.1053/j.otsm.2009.12.014

[2] Goutallier D, Bernageau J, Lecudonnec B (1978). The measurement of the tibial tuberosity. Patella groove distanced technique and results (author's transl). Rev Chir Orthop Reparatrice Appar Mot.

[3] Schoettle PB, Zanetti M, Seifert B, et al. (2006) The tibial tuberosity–trochlear groove distance; a comparative study between CT and MRI scanning. The Knee 13:26–31. 10.1016/j.knee.2005.06.003 10.1016/j.knee.2005.06.003 16023858

[4] Dejour H, Walch G, Nove-Josserand L, Guier C (1994). Factors of patellar instability: an anatomic radiographic study. Knee Surg Sports Traumatol Arthrosc.

[5] Schöttle PB, Fucentese SF, Pfirrmann C, Bereiter H, Romero J (2005). Trochleaplasty for patellar instability due to trochlear dysplasia: a minimum 2-year clinical and radiological follow-up of 19 knees. Acta Orthop.

[6] Diks MJF, Wymenga AB, Anderson PG (2003). Patients with lateral tracking patella have better pain relief following CT-guided tuberosity transfer than patients with unstable patella. Knee Surg Sports Traumatol Arthrosc.

[7] Hughes AJ, O'HEireamhoin S, Heffernan E, Hurson C (2015) A simple approach to assessment of a total knee replacement’s rotationary profile using computed tomography. Orthop Surg 7:350–353. 10.1111/os.12211 10.1111/os.12211 PMC6583725 26792396

[8] Schindelin J, Arganda-Carreras I, Frise E, Kaynig V, Longair M, Pietzsch T, Preibisch S, Rueden C, Saalfeld S, Schmid B, Tinevez J-Y, White DJ, Hartenstein V, Eliceiri K, Tomancak P, Cardona A (2012). Fiji: an open-source platform for biological-image analysis. Nat Methods.

